# Predisposition to Childhood Otitis Media and Genetic Polymorphisms within the Toll-Like Receptor 4 (*TLR4*) Locus

**DOI:** 10.1371/journal.pone.0132551

**Published:** 2015-07-15

**Authors:** Lena Hafrén, Elisabet Einarsdottir, Erna Kentala, Sari Hammarén-Malmi, Mahmood F. Bhutta, Carol J. MacArthur, Beth Wilmot, Margaretha Casselbrant, Yvette P. Conley, Daniel E. Weeks, Ellen M. Mandel, Outi Vaarala, Anna Kallio, Merit Melin, Janne K. Nieminen, Eira Leinonen, Juha Kere, Petri S. Mattila

**Affiliations:** 1 Department of Otorhinolaryngology, Helsinki University Central Hospital, Helsinki, Finland; 2 Department of Biosciences and Nutrition, Center for Innovative Medicine, Karolinska Institutet, Huddinge, Sweden; 3 UCL Ear Institute, London, United Kingdom; 4 Department of Otolaryngology, Head & Neck Surgery, Oregon Health & Science University, Portland, Oregon, United States of America; 5 Oregon Clinical and Translational Research Institute and Division of Bioinformatics and Computational Biology, Oregon Health & Science University, Portland, Oregon, United States of America; 6 Department of Otolaryngology, University of Pittsburgh School of Medicine, Pittsburgh, Pennsylvania, United States of America; 7 Department of Nursing and Human Genetics, University of Pittsburgh, Pittsburgh, Pennsylvania, United States of America; 8 Department of Human Genetics, Graduate School of Public Health, University of Pittsburgh, Pittsburgh, Pennsylvania, United States of America; 9 Institute of Clinical Medicine, University of Helsinki, Helsinki, Finland; 10 Department of Vaccination and Immune Protection, National Institute for Health and Welfare (THL), Helsinki, Finland; 11 Molecular Neurology Research Program, University of Helsinki and Folkhälsan Institute of Genetics, Helsinki, Finland; Kunming Institute of Zoology, Chinese Academy of Sciences, CHINA

## Abstract

**Background:**

Predisposition to childhood otitis media (OM) has a strong genetic component, with polymorphisms in innate immunity genes suspected to contribute to risk. Studies on several genes have been conducted, but most associations have failed to replicate in independent cohorts.

**Methods:**

We investigated 53 gene polymorphisms in a Finnish cohort of 624 cases and 778 controls. A positive association signal was followed up in a tagging approach and tested in an independent Finnish cohort of 205 cases, in a British cohort of 1269 trios, as well as in two cohorts from the United States (US); one with 403 families and the other with 100 cases and 104 controls.

**Results:**

In the initial Finnish cohort, the SNP rs5030717 in the *TLR4* gene region showed significant association (OR 1.33, *P* = .003) to OM. Tagging SNP analysis of the gene found rs1329060 (OR 1.33, *P* = .002) and rs1329057 (OR 1.29, *P* = .003) also to be associated. In the more severe phenotype the association was stronger. This finding was supported by an independent Finnish case cohort, but the associations failed to replicate in the British and US cohorts. In studies on TLR4 signaling in 20 study subjects, the three-marker risk haplotype correlated with a decreased TNFα secretion in myeloid dendritic cells.

**Conclusions:**

The *TLR4* gene locus, regulating the innate immune response, influences the genetic predisposition to childhood OM in a subpopulation of patients. Environmental factors likely modulate the genetic components contributing to the risk of OM.

## Introduction

Otitis media (OM) is the leading cause of doctor visits and antibiotic prescriptions in young children. An isolated episode of acute otitis media (AOM) is very common: up to 90% of all 3-year old children experience at least one episode, which usually resolves uneventfully [1]. However, approximately 10 to 15% of all children are otitis prone. They suffer from recurrent episodes of AOM (RAOM) and may have their first episode of AOM at a very early age. Childhood OM may also present as chronic otitis media with effusion (COME) which is characterized by indolent but prolonged inflammatory middle ear effusion (MEE) lasting for months and leading to conductive hearing loss [2,3].

Major risk factors for OM include environmental factors such as exposure to respiratory pathogens and passive smoking [4]. Although environmental factors clearly have a major role, genetic predisposition strongly influences the risk of OM. Studies of large twin cohorts in the United States (US) [5], United Kingdom (UK) [6], and Norway [7] have demonstrated that genetic factors are significantly associated with OM. We have recently shown a strong genetic component in the risk of OM in pedigrees within our Finnish cohort. The estimate of heritability was 39% for RAOM, 22% for COME, and 48% for all OM [8].

Genetic components predisposing to diseases are often evaluated using genome wide association studies that do not require prior assumptions of loci underlying disease susceptibility. Such studies have also been performed on OM, but many of these have suffered from a small sample size, and have failed to identify specific genes with a clear function in OM pathogenesis [9–13]. Another approach to identifying genetic components is to evaluate candidate genes with a plausible function in the pathogenesis of OM. OM candidate genes studies have mainly involved genes associated with innate immunity and inflammation [14]; they are reasonable targets for evaluation, as the initial development of OM likely involves a failure in the early steps of pathogen clearance. Previous candidate gene studies in OM have yielded encouraging results but most have not been replicated in independent cohorts [15,16].

To investigate the role of putative candidate genes in OM more thoroughly, we designed a study looking at previously reported genetic associations. We also included in our analysis polymorphisms implicated in the pathogenesis of asthma, as this shares with OM the characteristic of an inflammatory disease of the respiratory tract [17–20]. Our cohort of 624 Finnish otitis prone children and 778 blood donor control subjects is so far the largest cohort studied in an OM candidate gene study.

## Material and Methods

### Study subjects

The study subjects for the Finnish index cohort were recruited from patients who were referred to the Helsinki University Central Hospital due to RAOM or COME. The criteria for RAOM was >3 AOMs in 6 months or >4 AOMs in 12 months [21]. The criterion for COME was effusion in the middle ear for more than 2 months. We considered study subjects affected if they had RAOM or COME, or if they had had insertion of tympanostomy tubes.

Written informed consent was obtained from the children’s guardians. Information about the study subjects’ OM history, as well as medical history, and other relevant information was gathered as described previously [8]. DNA was extracted from peripheral blood using the FlexiGene DNA Kit (Qiagen, Hilden, Germany).

The Finnish index cohort consisted of 624 children from separate families, all suffering from RAOM (86%) or COME (68%). Most of the children had insertion of tympanostomy tubes (91%), some repeatedly. The mean number of tympanostomy tubes was 1.63. The study protocol was approved by the Ethics Committee at the Hospital District of Helsinki and Uusimaa and the study was conducted according to the Declaration of Helsinki ethical principles for medical research involving human subjects.

The independent Finnish replication cohort consisted of 205 1–4 year old children with RAOM (≥3 AOM /6 months or ≥5 AOM /12 months) and/or COME with no previous placement of tympanostomy tubes nor surgery on adenoids or tonsils [22].

Healthy Finnish blood donors (N = 778) were used as controls for the Finnish cohorts.

The UK cohort was recruited from UK patients undergoing tympanostomy tube insertions together with their family members during April 2009 to November 2013. COME (MEE ≥3 months) was confirmed by effusion at myringotomy and RAOM was assessed by clinical history using the same criteria as in the Finnish index cohort. A set of 1269 UK trios (parents and a child/children with OM), were included in the current study. DNA was extracted from saliva samples collected by Oragene OG-250 (DNA Genotek Inc., Kanata, Ontario, Canada) using an automated system (LGC Genomics, Hoddesdon, UK). Approval for the study was granted by the NHS Oxfordshire Research Ethics Committee (study reference 08/H0605/109). Written informed consent was obtained from the children’s guardians.

The Portland cohort (Oregon, USA) included children ages 18 months to 18 years undergoing tympanostomytube placement for COME (MEE > 3 months) [23]. Study subjects (100 patients with COME and 104 controls) were recruited and DNA extracted from saliva with the Oragene DNA Self-Collection Kit (DNA Genotek Inc., Kanata, Ontario, Canada). Approval from the Oregon Health and Science University institutional review board was obtained for the study. Written informed consent was obtained from the children’s guardians.

The Pittsburgh cohort was from the Children's Hospital of Pittsburgh, Pittsburgh, Pennsylvania, USA. The cohort consisted of 439 nuclear families containing 1,563 genotyped individuals. A subject was considered affected if he/she had undergone tympanostomy tube insertion at least once for RAOM and/or COME. DNA was isolated from blood using the Gentra PureGene method (Qiagen, Valencia CA). Approval was granted by the University of Pittsburgh Institutional Review Board. Written informed consent was obtained from the children’s guardians. [10]

### Genotyping and association analysis

Single nucleotide polymorphisms (SNPs) were selected in part from genes previously associated with RAOM and COME in candidate gene settings, and partly from SNPs associated with allergy and asthma in previous GWAS studies. We selected 53 SNPs in 35 genes for this analysis (S1 Table).

The genotyping of the Finnish index cohort was performed on the MassARRAY Platform from Sequenom at the MAF genotyping facility at Karolinska sjukhuset, Huddinge, Sweden. Six markers failed genotyping and three markers were monomorphic, leaving 44 markers for subsequent analysis and comprising 36 independent (r^2^ <0.4) markers. No SNPs showed significant (*P* < .05) deviation from Hardy–Weinberg equilibrium (HWE) in controls.

Allelic association of OM (or its sub-phenotypes) with each SNP was assessed using PLINK (v1.07). [24] Odds ratios (ORs) were calculated and statistical significance assessed using Fisher's exact test.

To verify our initial findings of association to a variant in *TLR4*, we set up a more detailed study of the *TLR4* region (S2 Table). Haploview 4.2 was used to select 16 *TLR4* tagging SNPs from a 140kB locus. This covers the whole linkage disequilibrium (LD) block within which the *TLR4* gene resides and a 97kB upstream and 32kB downstream region [25]. For the tagging strategy, SNPs with a minor allele frequency (MAF) >15% in the CEU population and r^2^<0.8 were selected. An additional set of four SNPs that have previously been associated with OM were selected [26], as well as two previously described *TLR4* coding variants (rs4986791 and rs4986790) [27]. Two SNPs failed assay design, leaving 20 SNPs in the follow-up study (S2 Table).

Genotyping of the *TLR4* tagging SNPs was performed as in the candidate gene study. Of the selected SNPs, one marker was excluded due to a call rate <90%, leaving 19 markers within the *TLR4* locus for analysis. No markers showed significant deviation from HWE in controls. Odds ratios were calculated and statistical significance assessed using Fisher's exact test.

The power of each cohort to identify a variant with 0.23 minor allele frequency, assuming a population prevalence of 10%, and an OR of 1.33, was assessed using the Genetic Power Calculator available at http://pngu.mgh.harvard.edu/~purcell/gpc/ [28]. These parameters are based on our data for rs1329060.

### *TLR4* replication studies

We attempted to validate our findings in independent datasets. We chose three markers for this validation, rs1329060, rs1329057, and rs5030717, which comprise the Finnish OM TCG risk haplotype.

The 205 children of the Finnish replication cohort were genotyped for the three risk haplotype-tagging markers using TaqMan SNP Genotyping Assays (Life Technologies, Thermo Fisher Scientific Inc., Waltham, MA, USA).

Study subjects from 1507 nuclear families (1269 full trios) of the UK replication cohort, as well as the Portland cohort were genotyped using KASP primer extension sequencing by LGC Genomics (http://www.lgcgroup.com/products/kasp-genotyping-chemistry/). Individuals with more than one missing genotype were excluded from the analysis, as well as families showing inheritance errors in any marker. PLINK 1.07 was used to assess transmission disequilibrium (TDT test) in trios.

The Pittsburgh cohort was genotyped using TaqMan allele discrimination assays (Life Technologies, Thermo Fisher Scientific Inc., Waltham, MA, USA). Duplicate samples across plates were used to detect plate to plate inconsistencies and independent double blind calls were made, with discrepancies reconciled by review of raw data or repeat-genotyping.

Unphased 3.1.7 (https://sites.google.com/site/fdudbridge/software/unphased-3-1) was used to test association in the Pittsburgh families [29].

### Comparison of OM risk in index and replication cohorts

ForestPlotViewer 1.1 (http://ntp.niehs.nih.gov/go/tools_forestplotviewer) and Adobe Illustrator CS6 were used to generate a forest plot, allowing for a visual overview of the association of each cohort to rs1329060.

### TNFα intracellular staining and mRNA analyses

For the functional studies of TLR4 signaling, we recruited ten age- and sex-matched pairs with risk (TCG) or protective (CTA) *TLR4* haplotypes included in the index cohort of 624 cases (S3 Table). Peripheral blood was taken from each pair on the same day and analyzed in parallel. Mononuclear cells were also isolated for mRNA analysis. FACS and mRNA studies on TLR4 proved to be complex, we therefore decided to use the downstream target TNFα as a proxy for the expression of the biologically functional TLR4 protein [30]. Protocols for fluorescence-activated cell sorting flow cytometry (FACS) preparation, mononuclear cell separation and mRNA work are detailed in S1 Data.

FACS results were analyzed with FlowJo 7.6.1 and GraphPad Prism 6. An unpaired, nonparametric Mann-Whitney test was used to test differences in subjects with the risk versus protective haplotype, as well as differences between all three genotypic groups.

### Analyses of gene expression databases

We used the FANTOM5 database (http://fantom.gsc.riken.jp/zenbu/) to look at transcription start sites and transcripts of *TLR4* and its downstream molecules, as well as expression levels in various cell/tissue types. We used the GTEx Portal (http://www.gtextportal.org) to look at potential expression quantitative trait loci (eQTLs) within the *TLR4* locus.

## Results

### Association in the Finnish cohort

Of the 53 polymorphisms investigated in the Finnish index cohort of 624 cases and 778 controls, one SNP, rs5030717 (A/G) located in the third intron of the *TLR4* gene, was significantly associated with risk of OM (risk minor allele G, OR = 1.33, *P* = .003) (Table 1, S4 Table). We subsequently performed a tagging SNP analysis of the *TLR4* gene region, revealing an additional association particularly at the SNPs rs1329060 and rs1329057 (OR = 1.33, *P* = .002 and OR 1.29, *P* = .003, respectively, S5 Table). These three SNPs are in strong LD with each other and thus define a putative *TLR4* haplotype associated with an increased risk of OM (S1 Fig). Other SNPs in the *TLR4* region also showed indications of association with otitis media, but it is unclear what the significance of these findings are and if these constitute an independent signal or not.

**Table 1:** Association analysis in the Finnish index cohort of 624 children with RAOM and/or COME, of the markers rs5030717, s1329060, and rs1329057 in the *TLR4*-region.

	SNP	A1	fA Cases	fA Controls	OR	95% CI	*P* Value (uncorr)
**ALL OM, N = 624**	rs5030717	G	0.23	0.18	1.33	1.10–1.61	.00329*
	rs1329060	T	0.28	0.23	1.33	1.11–1.58	.00157*
	rs1329057	C	0.31	0.26	1.29	1.09–1.53	.00296*
**COME, N = 536**	rs5030717	G	0.23	0.18	1.34	1.09–1.66	.00655
	rs1329060	T	0.29	0.23	1.37	1.13–1.66	.00160*
	rs1329057	C	0.33	0.26	1.36	1.13–1.64	.00115*
**RAOM, N = 422**	rs5030717	G	0.23	0.18	1.33	1.09–1.61	.00478*
	rs1329060	T	0.29	0.23	1.33	1.11–1.59	.00227*
	rs1329057	C	0.31	0.26	1.29	1.08–1.54	.00437
**Multiple insertions of tympanostomy tubes** ^**1**^ **, N = 203**	rs5030717	G	0.27	0.18	1.68	1.30–2.18	.00008*
	rs1329060	T	0.33	0.23	1.65	1.30–2.11	.00004*
	rs1329057	C	0.37	0.26	1.62	1.28–2.04	.00006*
**Early onset** ^**2**^ **, N = 32**	rs5030717	G	0.31	0.18	2.05	1.19–3.54	.00860
	rs1329060	T	0.42	0.23	2.42	1.45–4.04	.00048*
	rs1329057	C	0.44	0.26	2.19	1.32–3.63	.00201*

SNP = single nucleotide polymorphism, A1 = minor allele, fA = frequency of the minor allele, OR = odds ratio, All OM = all affected OM patients, COME = chronic otitis media with effusion, RAOM = recurrent otitis media.

*significant (*P* < .05) after permutation correction of 10000.

^1^ insertions of tympanostomy tubes ≥ 2.

^2^ First AOM before the age of six months.

There was a stronger association in children with a more severe phenotype, children who had OM onset before the age of 6 months (rs1329060 OR = 2.42, *P* = .0005, rs1329057 OR = 2.19, *P* = .002) or who had repeated insertion of tympanostomy tubes (rs1329060 OR = 1.65, *P* = .00004, rs1329057 OR = 1.62, *P* = .00006) (Table 1).

### Association analysis in replication cohorts

We next wanted to investigate whether the same trend could be seen in an unrelated Finnish cohort and if the association replicated in other populations. Analysis of an independent cohort of 205 otitis prone Finnish children showed that the allele frequencies of the three markers (s1329060 (28.9%), rs1329057 (31.7%), and rs5030717 (22.3%)) were similar to those in the index cohort and combining the two case cohorts yielded significant association to OM (s1329060 OR = 1.32, *P* = 0.002; rs1329057 OR = 1.30, *P* = 0.003; rs5030717 OR = 1.34, *P* = 0.002) (Table 2, S6 Table). However, analysis of 1269 predominantly white trios from UK, 439 white families from the US, and an independent cohort of 100 cases and 104 controls from the US failed to show any association between the three SNPs and risk of OM (Table 2, S6 Table). Of interest was that the frequencies of the minor alleles of the three SNPs in the Finnish population were much higher than in the UK and US populations.

**Table 2:** Replication analysis of SNPs in the *TLR4* region in a UK cohort and in two US cohorts. For comparison, the allele frequencies from an independent Finnish case-cohort are shown.

	**N Trios**		**SNP**	**A1**	**Trans**	**Untrans**	**OR**	**95% CI**	***P* value**
**UK**	1269								
			rs1329060	T	273	274	1.00	0.84–1.17	.966
			rs1329057	C	347	349	0.99	0.86–1.15	.940
			rs5030717	G	222	214	1.04	0.86–1.25	.702
**Pittsburgh, US**	439								
			rs1329060	T	136	182	0.79	0.62–1.00	.050
			rs1329057	C	168	212	0.85	0.69–1.06	.158
			rs5030717	G	118	141	0.94	0.72–1.22	.654
	**N Case**	**N Control**	**SNP**	**A1**	**fA Case**	**fA Control**	**OR**	**95% CI**	***P* value**
**Portland, US**	100	104							
			rs1329060	T	0.085	0.148	0.53	0.28–1.04	.059
			rs1329057	C	0.125	0.185	0.63	0.35–1.32	.113
			rs5030717	G	0.061	0.099	0.59	0.27–1.28	.179
**Finnish replication cohort**	205								
			rs1329060	T	0.289				
			rs1329057	C	0.317				
			rs5030717	G	0.223				

N = Number, SNP = single nucleotide polymorphism, A1 = minor allele, fA = frequency of the minor allele, OR = odds ratio, trans/untrans = transmitted/untransmitted allele in the TDT-test, US = United States, UK = United Kingdom.

**Fig 1:**
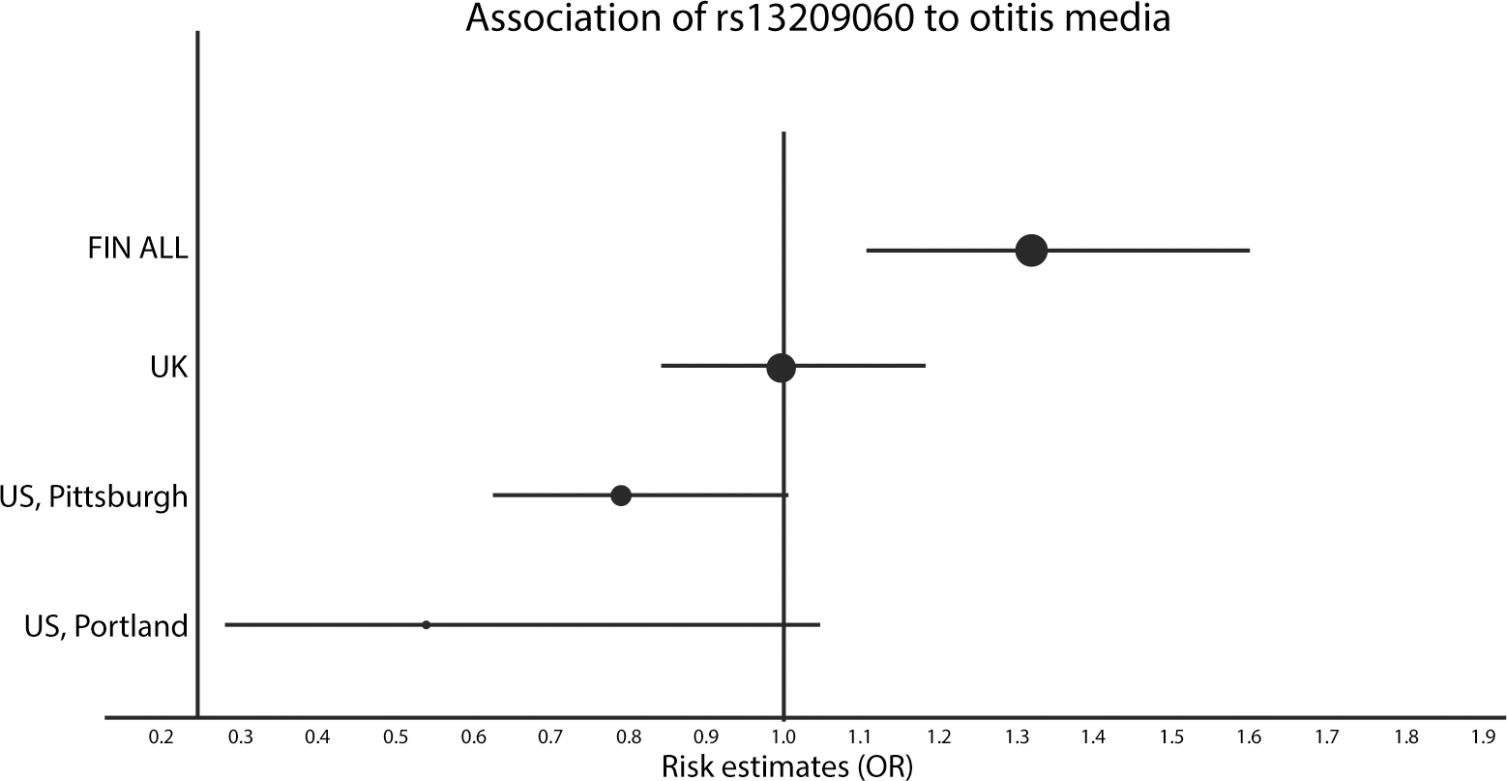
Comparison of the *TLR4* variant rs13209060 association to otitis media. The size of the circle has been scaled to show the relative sizes of each dataset (N = number of cases in case-control datasets or N = twice the number of probands in family-based analysis). Horizontal lines have been scaled to show the 95% confidence intervals (95% CI) of the odds ratios of the T-allele, the position of the circles on the x-axis shows the odds ratio for that dataset. For simplicity and to highlight the result with the strongest signal in the Finnish population, we only plotted results of the association of rs13209060-T with otitis media; additional data can be found in S6 Table.

The comparison of association between rs1329060-T and OM in all four cohorts is shown in Fig 1. While the Finnish dataset shows a clear indication of rs1329060-T conferring risk of OM, other datasets either show no association or a tendency for the rs1329060-T allele to confer protection.

The Finnish index cohort, with 624 cases and 778 controls, had an estimated power of 76% to identify a variant with similar frequency and OR as the rs1329060 variant. The combined Finnish cohorts (829 cases and 778 controls) had 82% power. The 1269 UK trios were estimated to have a 90% power to identify this variant. The two smaller US cohorts from Portland and Pittsburgh (100 cases/104 controls and 439 trios families, respectively), had 17% and 47% power.

### Functional studies in the Finnish index cohort

Looking at the *TLR4* locus through the GTEx portal (Figure A in S1 Fig) revealed a set of markers with a strong eQTL effect on *TLR4* expression (P <10^−10^) (Figure B in S1 Fig). The top seven of these markers share very similar minor allele frequencies (Figure B in S1 Fig) to rs1329060 and rs1329057, the two markers most strongly associated with OM in our study. One of these is rs2770146, a marker in strong LD with rs1329060 and rs1329057 (Figure C in S1 Fig), and showing strong eQTL effects with *TLR4* (Figure D in S1 Fig). The minor allele of each of the seven markers at GTEx correlates with lower expression of TLR4.

**Fig 2:**
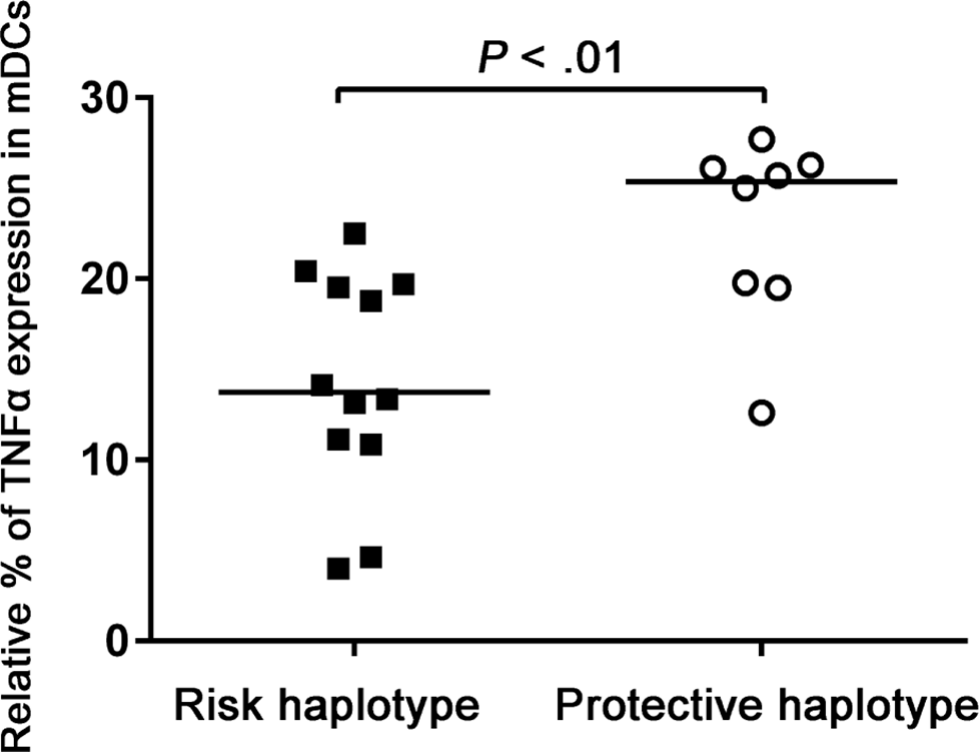
Expression of TNFα in myeloid dendritic cells from children with recurrent episodes of acute otitis media or chronic otitis media with effusion. The percentage of TNFα expressing cells in the unstimulated sample was subtracted from the percentage of the cells seen in the LPS stimulated sample. Children with the otitis media risk rs1329060-rs1329057-rs5030717 TCG *TLR4* gene haplotype were compared to children with the protective CTA haplotype by Mann-Whitney U-test.

**Fig 3:**
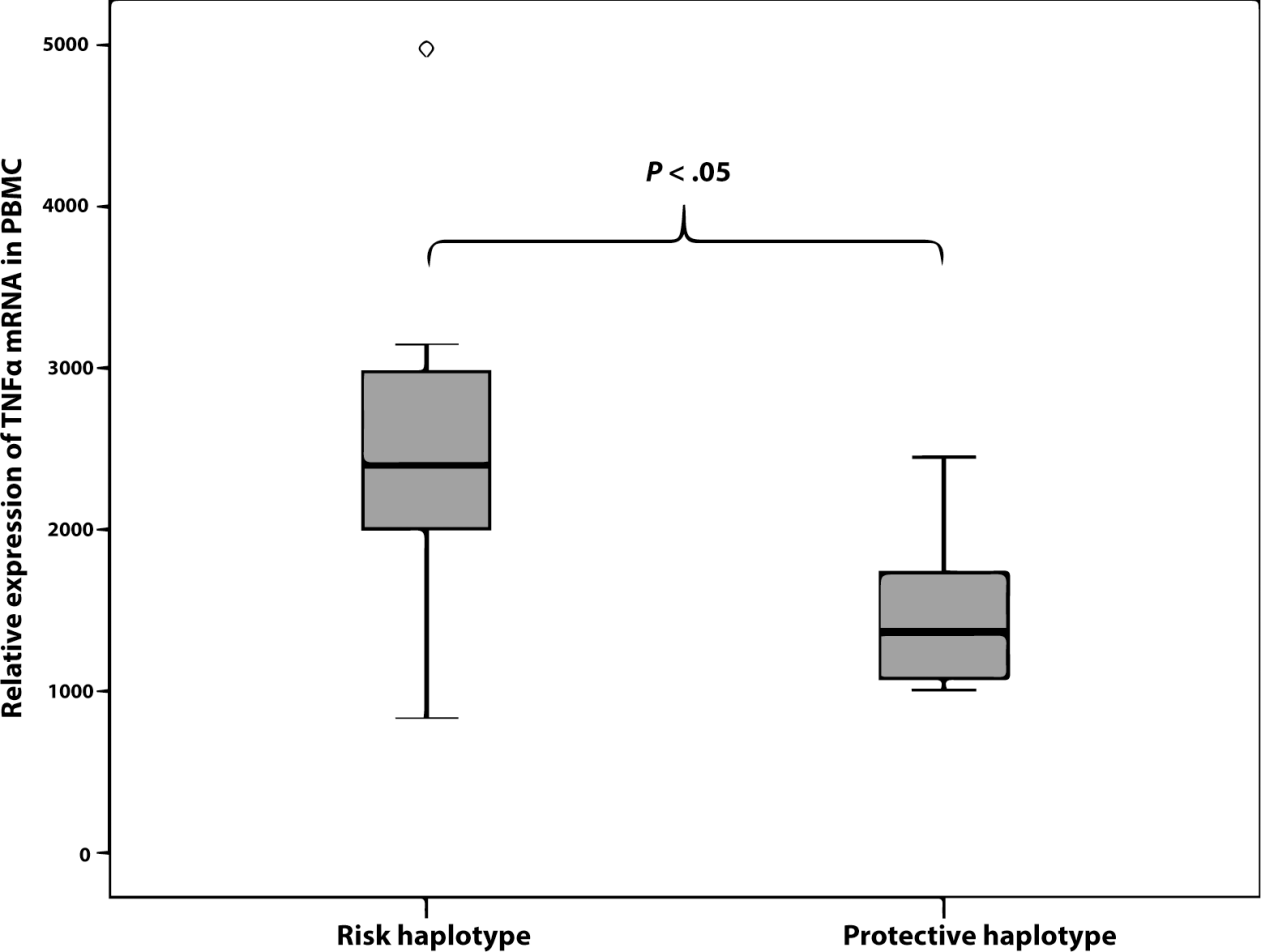
Relative mRNA expression of TNFα analyzed by RT-qPCR in peripheral blood mononuclear cells (PBMC) stimulated with LPS in subjects with protective rs1329060-rs1329057-rs5030717 CTA or risk TCG *TLR4* haplotype. The statistical difference between groups was assessed using the Mann-Whitney U-test. The expression level is shown as relative units calculated by the ddCT-method. Boxes indicate interquartile range (25%–75%) with the horizontal bar within the box indicating the median. Whiskers show minimum and maximum values, o = outlier.

Our OM TCG risk haplotype lies within a region of strong LD that covers the *TLR4* gene and its transcription starting site. We wanted to investigate whether the different OM *TLR4* haplotypes would convey functional characteristics determined in peripheral blood. Analysis of non-carriers and carriers of the *TLR4* TCG risk haplotype showed that carriers of the risk haplotype had significantly (*P* = .0073) lower expression of Tumor necrosis factor alpha (TNFα) protein as assessed by flow cytometry after stimulation with the TLR4 ligand lipopolysaccharide (LPS) in myeloid dendritic cells (Fig 2). We also observed changes in *TNFα* mRNA levels after LPS stimulation of peripheral blood mononuclear cells (PBMC) as assessed by quantitative polymerase chain reaction (qPCR) (Fig 3). Patients with the TCG risk haplotype had a higher relative expression of TNFα compared to the protective haplotype. The apparent discrepancy in these two methods may reflect the complex regulation of *TNFα* expression, which may result from a multitude of mechanisms including the multiple transcription start sites of *TNFα* mRNA (according to FANTOM5). Our qPCR measured only changes in the overall expression of a short segment of *TNFα* mRNA, most likely not truly reflecting overall expression of TNFα protein.

## Discussion

Our analysis of 35 candidate genes in 624 cases and 778 controls identified strong association of the *TLR4* gene region with risk of childhood OM in Finnish patients. A detailed analysis of the *TLR4* gene region identified a novel and previously unrecognized risk haplotype in Finnish OM cases, defined by the minor alleles of rs13209060, rs13209057, and rs5030717. The association was strongest in children who had their first OM episode at younger than 6 months and in children who had undergone multiple tympanostomy tube insertions. This suggested that the more severe phenotype of OM has the strongest connection with the *TLR4* TCG risk haplotype, which further supported a role of *TLR4* in OM pathogenesis. The *P* value of the association in the subset of patients with multiple tube insertions was of the order of 10^−5^, which is one of the most significant associations so far reported in OM candidate gene studies. An independent cohort of 205 Finnish otitis media prone children showed a similar trend.

In contrast, we did not find association between OM and other polymorphisms, including other *TLR4* SNPs previously associated with COME/RAOM (S4 Table, S5 Table) [15,16]. Differences in environmental factors or criteria of OM diagnosis may contribute to these discrepancies. Furthermore, we saw no significant association to SNPs previously implicated in association with asthma or other atopic diseases.

We were unable to replicate the association with *TLR4* in three independent OM cohorts in UK, Pittsburgh and Portland. One possible explanation may be that OM is a heterogeneous disease and that environmental factors modulate which specific genetic factors contribute to the overall predisposition to OM. Such environmental factors may include differences in vaccination policy, as the Finnish children were not vaccinated against pneumococci at the time they were recruited. Another issue that may contribute to apparent discrepancy is that the frequency of the *TLR4* TCG risk haplotype is 1.9 to 3.3 times higher in the Finnish population when compared to the frequency in UK and the US. In Finland some genetic variations have been enriched while others are much less common due to its population history [31] and the high frequency of the *TLR4* TCG risk haplotype in Finland may make its genetic contribution easier to detect. Finally, the size of the datasets influences the power to replicate the initial association. In particular the Portland cohort is quite small. Findings from these smaller datasets are potentially interesting, but should be interpreted with caution.

The functional studies on 20 study subjects revealed that carriers of the *TLR4* TCG risk haplotype had attenuated TNFα protein production in myeloid dendritic cells as response to LPS stimulation when compared to non-risk haplotypes. When TNFα cytokine mRNA production after LPS stimulation was evaluated in PBMCs, we found in contrast an increased up-regulation in carriers of the *TLR4* TCG risk haplotype. *TNFα* has several transcription start sites and even an anti-sense transcription start site 3’ of the gene [32], making direct comparisons between the TNFα protein and mRNA levels problematic. The change in mRNA levels being opposite when compared to the TNFα protein level is thus not necessarily contradictory, as regulation of mRNA and protein levels are complex.

The difference in TNFα protein expression between carriers of different *TLR4* haplotypes suggested that this difference would have been accompanied by a difference in TLR4 expression. We attempted to study this, but because the expression of TLR4 protein is weak [30], we were unable to get accurately quantifiable measurements of TLR4 surface expression by FACS. Measurement of *TLR4* mRNA is also challenging, as there are several different *TLR4* splice variants (http://genome.ucsc.edu) all of which do not code for a full-length protein and the function of which are poorly known. Nevertheless, the *TLR4* mRNA eQTL signal identified through the GTEx portal overlaps with the region containing our putative TCG risk haplotype. The markers within the region are in strong LD, and the eQTL signal is consistent with this. The direction of the eQTL signal also fits with our hypothesis of impaired TLR4 expression/signaling in OM patients, as well as our finding of a minor frequency haplotype conferring disease risk. It is plausible to assume that our associated markers are tagging a functional haplotype with real eQTL effects.

There may have been evolutionary pressure to conserve a low response haplotype, as other polymorphisms at the *TLR4* locus are also thought to be under evolutionary pressure [33]. Although the TCG haplotype described in this report may increase risk of OM, such a low response haplotype may be beneficial in other contexts. Vigorous responses through TLR4 may result in harmful outcomes, such as excessive inflammatory responses during sepsis, tissue injury, and allergic diseases [34–39]. Taken together, these multiple lines of evidence argue that the *TLR4* TCG risk haplotype has unique functional characteristics that may contribute to the risk of OM.

TLR4 presumably plays a major role in innate immunity against the pathogens causing OM. The major microbial ligand of TLR4 is LPS, a bacterial cell wall component of Gram-negative bacteria. TLR4 also recognizes other microbial antigens such as pneumococcal pneumolysin [40]. In addition, TLR4 has a role in the up-regulation of TLR2 [41], which recognizes the peptidoglycan of Gram-positive bacteria. TLR4 is thus important for the innate immune response against the major pathogens causing OM: the Gram-negative *Haemophilus influenzae* and *Moraxhella catarrhalis* and the Gram-positive *Streptococcus pneumoniae*. TLR4 also recognizes endogenous ligands such as heat shock proteins and proteolytically cleaved fibrinogen [42], which may potentially contribute to the pathogenesis of OM. Of note is that *Tlr4* knock-out mice have decreased pathogen clearance and prolonged inflammation in the middle ear during experimental OM [43], further supporting a connection between *TLR4* and OM risk.

We report here a strong association between a previously unrecognized *TLR4* haplotype and risk of OM in two independent Finnish cohorts and show that this haplotype correlates with unique functional characteristics. We were unable to replicate this association in a UK cohort or in two US cohorts. This may reflect the heterogeneous nature of OM, and that interaction of environmental and host factors may modulate which genetic factors contribute to overall predisposition to OM. Such heterogeneity of OM may result from the magnitude and quality of exposure to respiratory microbes. Heterogeneity of OM in different populations poses a challenge in future studies of OM genetics.

## Supporting Information

S1 Data Laboratory protocols. (DOCX)

S2 Data Original genotyping data for included study cohorts. (ZIP)

S1 Fig TLR4 eQTLs, visualized through the GTEx portal. (TIF)

S1 Table Single nucleotide polymorphisms studied in candidate gene study in Finnish index cohort of 624 children with RAOM and/or COME. (DOCX)

S2 Table SNPs for verification of *TLR4* results in the Finnish index cohort of 624 children with RAOM and/or COME. (DOCX)

S3 Table Study subjects for the functional studies. 20 Finnish age- and sex- matched patients with RAOM and/or COME.
(DOCX)

S4 Table Genotyping results for candidate gene study in the Finnish index cohort of 624 affected children and 778 healthy controls. (DOCX)

S5 Table Follow-up study on *TLR4* in the Finnish index cohort of 624 affected children with RAOM and/or COME and 778 healthy controls. (DOCX)

S6 Table A comparison of *TLR4* association to otitis media. (DOCX)
