# Supplementary material for: Predisposition to Childhood Otitis Media and Genetic Polymorphisms within the Toll-Like Receptor 4 (TLR4) Locus
Source: PLoS One. 2015 Jul 15;10(7):e0132551. doi: 10.1371/journal.pone.0132551 (PMC4503307; doi:10.1371/journal.pone.0132551)
Supplement: S1 Data — (DOCX) [file pone.0132551.s001.docx]

**Data S1** Laboratory protocols

**TNFα intracellular staining in 20 Finnish patients with RAOM and/or COME**

Fresh heparinized blood was stimulated with the TLR4 ligand LPS (1μg/ml, Sigma-Aldrich, NY) for a total of 5 hours. Monensin (BD GolgiStop, BD Biosciences, CA) was added after 1 hour of stimulation. Cells were stained with fluorescence conjugated antibodies against CD14 (PE-Cy5.5, Invitrogen, MHCD1418), CD1c (APC, Miltenyi, 130-090-903), CD11c (PE, BD Biosciences, 333149), and CD19 (PE-Cy5.5, Invitrogen, MHCD1918). Red blood cells were lysed using FACS Lysing solution (BD Biosciences, 349202). After centrifugation (500g for 5min) and washing (5% FBS+0.02% NaN_3_ in PBS), the cells were permeabilized with Cytofix/Cytoperm Plus Fixation and Permeabilization Kit (BD Biosciences, 555028). The cells were then stained either with anti-TNFα PE antibody (BD Pharmingen, 554513) or an isotype control (mouse IgG1, BD Pharmingen, 554680). Unbound antibodies were washed, cells were re-suspended in paraformaldehyde (1%), stored at +4°C, and analyzed with FACSCalibur flow cytometer (BD Biosciences) the following day.

**Method for separation of peripheral blood mononuclear cells and RNA extraction**

Peripheral blood mononuclear cells were isolated from EDTA blood using density centrifugation (Ficoll-Paque, Amersham Biosciences, Uppsala, Sweden). Isolated mononuclear cells were suspended in cell culture media consisting of RPMI 1640 (Gibco, ref. 42401) supplemented with 5% heat-inactivated human AB-serum (Innovative research), 2mmol/l L-glutamine (Gibco), and 25μg/ml Gentamicin (Sigma). The cells were frozen in the above media supplemented with 10% DMSO (Sigma, D2650) at 3 to 5 million cells /ml.

After thawing, peripheral blood mononuclear cells were stimulated in the above cell culture media with or without LPS (1 μg/ml, Sigma) for 4 hours. Total RNA was isolated with Qiagen RNeasy Mini kit (Qiagen, Hilden Germany) and cDNA was synthesized using High Capacity cDNA Reverse Transcription kit (Applied Biosystems, Foster City, CA). Gene expression levels were analyzed with real-time quantitative polymerase chain reaction (qPCR) using TaqMan Gene Expression assay specific for TNFα (Applied Biosystems, Foster City, CA, Hs00174128_m1). The qPCR runs were performed in triplicates and qPCR for ribosomal 18s subunit (Hs03928985_g1) was used as an endogenous control for normalization of the amount of RNA. We used a control RNA sample prepared from phytohaemagglutinin stimulated human peripheral blood mononuclear cells to control for inter-assay variation.
