# Supplementary figures and images for: Predisposition to Childhood Otitis Media and Genetic Polymorphisms within the Toll-Like Receptor 4 (TLR4) Locus
Source: PLoS One. 2015 Jul 15;10(7):e0132551. doi: 10.1371/journal.pone.0132551 (PMC4503307; doi:10.1371/journal.pone.0132551)

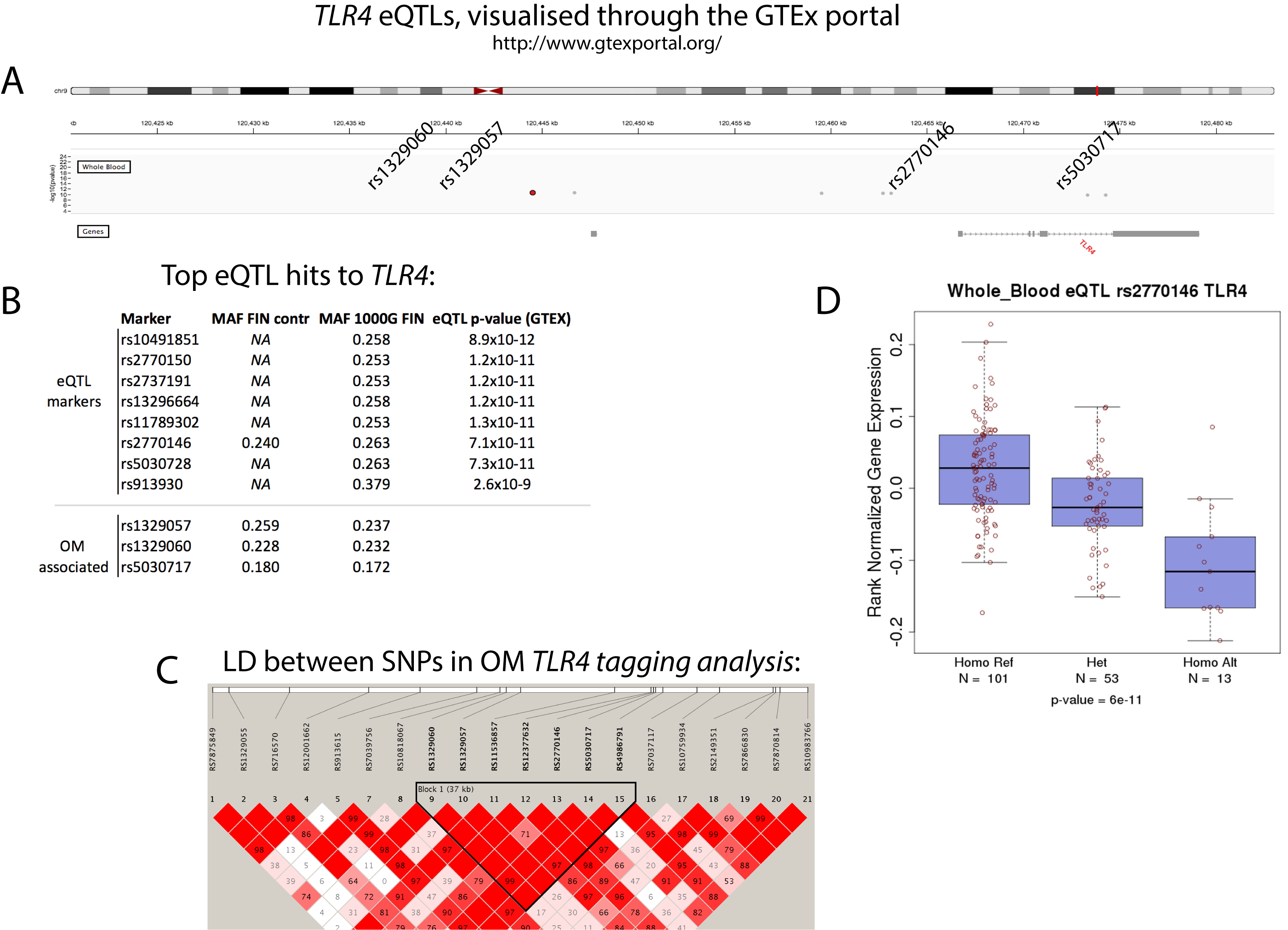

Supplement: S1 Fig — (TIF) [file pone.0132551.s003.tif]
