# Supplementary material for: Predisposition to Childhood Otitis Media and Genetic Polymorphisms within the Toll-Like Receptor 4 (TLR4) Locus
Source: PLoS One. 2015 Jul 15;10(7):e0132551. doi: 10.1371/journal.pone.0132551 (PMC4503307; doi:10.1371/journal.pone.0132551)
Supplement: S1 Table — (DOCX) [file pone.0132551.s004.docx]

**Table S1** Single nucleotide polymorphisms studied in candidate gene study in Finnish index cohort of 624 children with RAOM and/or COME. MAF = minor allele frequency according to CEU population in HapMap Release #24, phase II.

| **Gene** | **SNP** | **Associated to** | **MAF** | **Chromosome** | **Reference** |
| --- | --- | --- | --- | --- | --- |
| CD14 | rs2569190 | Otitis media | A=0.474 | 5q31.3 | [1, 2] |
| FBXO11 | rs2134056 | Otitis media | T=0.113 | 2p16.3 | [3, 4] |
| FBXO11 | rs330787 | Otitis media | A=0.351 | 2p16.3 | [3, 4] |
| FCGR2a | rs1801274 | Otitis media | G=0.420 | 1q23.3 | [5] |
| IL10 | rs1554286 | Otitis media | A=0.381 | 1q31-q32 | [6] |
| IL10 | rs1800871 | Otitis media | A=0.420 | 1q32.1 | [7] |
| IL10 | rs1800872 | Otitis media | T=0.422 | 1q32.1 | [7] |
| IL10 | rs1800890 | Otitis media | T=0.226 | 1q31-q32 | [6] |
| IL10 | rs1800896 | Otitis media | C=0.297 | 1q32.1 | [8] |
| IL10 | rs3021094 | Otitis media | G=0.155 | 1q31-q32 | [6] |
| IL1A | rs1800587 | Otitis media | A=0.258 | 2q13 | [9] |
| MBL2 | rs11003125 | Otitis media | C=0.319 | 10q21.1 | [10] |
| MBL2 | rs1800451 | Otitis media | T=0.064 | 10q21.1 | [10] |
| MBL2 | rs7096206 | Otitis media | G=0.182 | 10q21.1 | [10] |
| MBL2 | rs930507 | Otitis media | G=0.247 | 10q21.1 | [10] |
| FBXO11 | rs2020911 | Otitis media | T=0.372 | 2p16.4 | [4] |
| MUC5B | rs2735733 | Otitis media | T=0.475 | 11p15.5 | [6] |
| SMAD2 | rs1792658 | Otitis media | C=0.310 | 18q21.1 | [3] |
| TLR4 | rs5030717 | Otitis media | G=0.101 | 9q33.1 | [11] |
| TNFA | rs1800629 | Otitis media | A=0.096 | 6p21.33 | [7, 12, 13] |
| TNFA | rs1800750 | Otitis media | A=0.013 | 6p21.33 | [8] |
| TNFA | rs361525 | Otitis media | A=0.046 | 6p21.33 | [8] |
| C11orf30 | rs7927894 | Atopic dermatitis | T=0.283 | 11q13.5 | [14] |
| C6orf10 | rs3129943 | Asthma | G=0.242 | 6p21.32 | [15] |
| CDK2 | rs2069408 | Asthma | G=0.258 | 12q13.2 | [15] |
| CTNNA3 | rs10762058 | Asthma | C=0.212 | 10q21.3 | [16] |
| DENND1B, CRB1 | rs2786098 | Asthma | T=0.183 | 1q31.3 | [17] |
| GSDMA | rs3894194 | Asthma | A=0.398 | 17q21.1 | [18] |
| GSDMB | rs11078927 | Asthma | T=0.467 | 17q12 | [19] |
| GSDMB | rs2305480 | Asthma | A=0.332 | 17q12 | [18] |
| HLA-DQ | rs9273349 | Asthma | NA | 6p21.32 | [18] |
| IKZF4 | rs1701704 | Asthma | C=0.292 | 12q13.2 | [15] |
| IL18R1 | rs3771166 | Asthma | A=0.400 | 2q12.1 | [18] |
| IL2RB | rs2284033 | Asthma | A=0.443 | 22q12.3 | [18] |
| KIAA1271 | rs4815617 | Asthma | T=0.055 | 20p13 | [20] |
| LOC338591 | rs10508372 | Asthma | A=0.271 | 10p14 | [15] |
| NPSR1 | rs323917 | Asthma and allergy | G=0.049 | 7p14.3 | [21] |
| NPSR1 | rs323922 | Asthma and allergy | C=0.476 | 7p14.3 | [21] |
| NPSR1 | rs324377 | Asthma and allergy | A=0.498 | 7p14.3 | [21] |
| NPSR1 | rs324384 | Asthma and allergy | C=0.440 | 7p14.3 | [21] |
| NPSR1 | rs324396 | Asthma and allergy | T=0.285 | 7p14.3 | [21] |
| NPSR1 | rs740347 | Asthma and allergy | T=0.285 | 7p14.3 | [21] |
| ORMDL3 | rs6503525 | Asthma | C=0.432 | 17q12 | [22] |
| PDE4D | rs1588265 | Asthma | G=0.375 | 5q12.1 | [23] |
| PYHIN1 | rs1101999 | Asthma | C=0.072 | 1q23.1 | [15] |
| RAD50 | rs2244012 | Asthma | G=0.328 | 5q31.1 | [20] |
| RORA | rs11071559 | Asthma | T=0.252 | 15q22.2 | [18] |
| SGK493 | rs4952590 | Atopic dermatitis | T=0.265 | 2p21 | [24] |
| SLC22A5 | rs2073643 | Asthma | C=0.404 | 5q31.1 | [18] |
| SLC30A8 | rs3019885 | Asthma | G=0.344 | 8q24.11 | [25] |
| SMAD3 | rs744910 | Asthma | A=0.452 | 15q22.33 | [18] |
| TLE4, CHCHD9 | rs2378383 | Asthma | G=0.120 | 9q21.31 | [26] |
| Intergenic | rs9319321 | Asthma | A=0.245 | 13q12.13 | [16] |

**References for Table S1**

1. Wiertsema SP, Khoo SK, Baynam G, et al. Association of CD14 promoter polymorphism with otitis media and pneumococcal vaccine responses. Clin Vaccine Immunol, **2006**; 13(8): 892-7.

2. Carroll SR, Zald PB, Soler ZM, Milczuk HA, Trune DR, Macarthur CJ. Innate immunity gene single nucleotide polymorphisms and otitis media. Int J Pediatr Otorhinolaryngol, **2012**.

3. Rye MS, Wiertsema SP, Scaman ES, et al. FBXO11, a regulator of the TGFbeta pathway, is associated with severe otitis media in western australian children. Genes Immun, **2011**; 12(5): 352-9.

4. Segade F, Daly KA, Allred D, et al. Association of the FBXO11 gene with chronic otitis media with effusion and recurrent otitis media: The minnesota COME/ROM family study. Arch Otolaryngol Head Neck Surg, **2006**; 132(7): 729-33.

5. Wiertsema SP, Veenhoven RH, Walraven V, et al. Pneumococcal vaccine efficacy for mucosal pneumococcal infections depends on fcgamma receptor IIa polymorphism. Vaccine, **2006**; 24(6): 792-7.

6. Sale MM, Chen WM, Weeks DE, et al. Evaluation of 15 functional candidate genes for association with chronic otitis media with effusion and/or recurrent otitis media (COME/ROM). PLoS One, **2011**; 6(8): e22297.

7. Alper CM, Winther B, Hendley JO, Doyle WJ. Cytokine polymorphisms predict the frequency of otitis media as a complication of rhinovirus and RSV infections in children. Eur Arch Otorhinolaryngol, **2009**; 266(2): 199-205.

8. Emonts M, Veenhoven RH, Wiertsema SP, et al. Genetic polymorphisms in immunoresponse genes TNFA, IL6, IL10, and TLR4 are associated with recurrent acute otitis media. Pediatrics, **2007**; 120(4): 814-23.

9. Joki-Erkkila VP, Puhakka H, Hurme M. Cytokine gene polymorphism in recurrent acute otitis media. Arch Otolaryngol Head Neck Surg, **2002**; 128(1): 17-20.

10. Wiertsema SP, Herpers BL, Veenhoven RH, et al. Functional polymorphisms in the mannan-binding lectin 2 gene: Effect on MBL levels and otitis media. J Allergy Clin Immunol, **2006**; 117(6): 1344-50.

11. Sale M, Marion M, Perlegas P, et al. Comprehensive evaluation of 16 functional candidate genes for chronic otitis media with effusion And/or recurrent otitis media (COME/ROM) In: Santi PA, editor. Abstracts of the thirty-first annual midwinter research meeting of the association for research in otolaryngology; February 16-21, 2008; Phoenix, Arizona, USA. Association for Research in Otolaryngology, **2008**:.

12. Patel JA, Nair S, Revai K, et al. Association of proinflammatory cytokine gene polymorphisms with susceptibility to otitis media. Pediatrics, **2006**; 118(6): 2273-9.

13. Revai K, Patel JA, Grady JJ, Nair S, Matalon R, Chonmaitree T. Association between cytokine gene polymorphisms and risk for upper respiratory tract infection and acute otitis media. Clin Infect Dis, **2009**; 49(2): 257-61.

14. Esparza-Gordillo J, Weidinger S, Folster-Holst R, et al. A common variant on chromosome 11q13 is associated with atopic dermatitis. Nat Genet, **2009**; 41(5): 596-601.

15. Hirota T, Takahashi A, Kubo M, et al. Genome-wide association study identifies three new susceptibility loci for adult asthma in the japanese population. Nat Genet, **2011**; 43(9): 893-6.

16. Kim SH, Cho BY, Park CS, et al. Alpha-T-catenin (CTNNA3) gene was identified as a risk variant for toluene diisocyanate-induced asthma by genome-wide association analysis. Clin Exp Allergy, **2009**; 39(2): 203-12.

17. Sleiman PM, Flory J, Imielinski M, et al. Variants of DENND1B associated with asthma in children. N Engl J Med, **2010**; 362(1): 36-44.

18. Moffatt MF, Kabesch M, Liang L, et al. Genetic variants regulating ORMDL3 expression contribute to the risk of childhood asthma. Nature, **2007**; 448(7152): 470-3.

19. Torgerson DG. Ampleford EJ. Chiu GY. et al. Meta-analysis of genome-wide association studies of asthma in ethnically diverse north american populations. Nat Genet, **2011**; 43(9): 887-92.

20. Li X, Howard TD, Zheng SL, et al. Genome-wide association study of asthma identifies RAD50-IL13 and HLA-DR/DQ regions. J Allergy Clin Immunol, **2010**; 125(2): 328,335.e11.

21. Laitinen T, Polvi A, Rydman P, et al. Characterization of a common susceptibility locus for asthma-related traits. Science, **2004**; 304(5668): 300-4.

22. Ferreira MA, McRae AF, Medland SE, et al. Association between ORMDL3, IL1RL1 and a deletion on chromosome 17q21 with asthma risk in australia. Eur J Hum Genet, **2011**; 19(4): 458-64.

23. Himes BE, Hunninghake GM, Baurley JW, et al. Genome-wide association analysis identifies PDE4D as an asthma-susceptibility gene. Am J Hum Genet, **2009**; 84(5): 581-93.

24. Castro-Giner F, Bustamante M, Ramon Gonzalez J, et al. A pooling-based genome-wide analysis identifies new potential candidate genes for atopy in the european community respiratory health survey (ECRHS). BMC Med Genet, **2009**; 10: 128.

25. Noguchi E, Sakamoto H, Hirota T, et al. Genome-wide association study identifies HLA-DP as a susceptibility gene for pediatric asthma in asian populations. PLoS Genetics, **2011**; 7(7): e1002170.

26. Hancock DB, Romieu I, Shi M, et al. Genome-wide association study implicates chromosome 9q21.31 as a susceptibility locus for asthma in mexican children. PLoS Genet, **2009**; 5(8): e1000623.
