# Supplementary material for: Predisposition to Childhood Otitis Media and Genetic Polymorphisms within the Toll-Like Receptor 4 (TLR4) Locus
Source: PLoS One. 2015 Jul 15;10(7):e0132551. doi: 10.1371/journal.pone.0132551 (PMC4503307; doi:10.1371/journal.pone.0132551)
Supplement: S2 Table — (DOCX) [file pone.0132551.s005.docx]

**Table S2** SNPs for verification of *TLR4* results in the Finnish index cohort of 624 children with RAOM and/or COME. MAF = minor allele frequency according to CEU population in HapMap Release #28.

| **SNP** | **Position**  **(b37)** | **MAF** | **Allele** | **Comment** | **Reference** |
| --- | --- | --- | --- | --- | --- |
| rs10759934 | 120488996 | 0.500 | T:A | tagging |  |
| rs10818067 | 120437061 | 0.183 | A:T | tagging |  |
| rs10983766 | 120509848 | 0.454 | C:G | tagging |  |
| rs11536857 | 120464136 | 0.054 | C:T | OM | [1] |
| rs12001662 | 120406009 | 0.415 | C:T | tagging |  |
| rs12377632 | 120472730 | 0.395 | T:C | OM | [1] |
| rs1329055 | 120372882 | 0.195 | C:T | tagging |  |
| rs1329057 | 120441946 | 0.217 | T:C | tagging |  |
| rs1329060 | 120438477 | 0.146 | C:T | tagging |  |
| rs1329066 | 120418308 | 0.167 | A:G | tagging |  |
| rs2149351 | 120501644 | 0.235 | G:T | tagging |  |
| rs2770146 | 120473338 | 0.331 | T:C | OM | [1] |
| rs4986791 | 120475602 | 0.031 | C:T | coding |  |
| rs7037117 | 120483663 | 0.208 | A:G | tagging |  |
| rs7039756 | 120431545 | 0.232 | A:C | tagging |  |
| rs716570 | 120387228 | 0.146 | G:A | tagging |  |
| rs7866830 | 120502265 | 0.177 | G:A | tagging |  |
| rs7870814 | 120503274 | 0.451 | G:A | tagging |  |
| rs7875849 | 120369028 | 0.211 | A:G | tagging |  |
| rs913615 | 120418118 | 0.473 | A:C | tagging |  |

**Reference for Table S2**

1. Sale M, Marion M, Perlegas P, et al. Comprehensive evaluation of 16 functional candidate genes for chronic otitis media with effusion and/or recurrent otitis media (COME/ROM), Assoc. Res. Otolaryngol. Abs.: 8.
