# Supplementary material for: Predisposition to Childhood Otitis Media and Genetic Polymorphisms within the Toll-Like Receptor 4 (TLR4) Locus
Source: PLoS One. 2015 Jul 15;10(7):e0132551. doi: 10.1371/journal.pone.0132551 (PMC4503307; doi:10.1371/journal.pone.0132551)
Supplement: S3 Table — 20 Finnish age- and sex- matched patients with RAOM and/or COME. (DOCX) [file pone.0132551.s006.docx]

**Table S3** Study subjects for the functional studies. 20 Finnish age- and sex- matched patients with RAOM and/or COME.

| **ID** | **Sex** | **Age** | **Genotype for the *TLR4* (TCG) risk haplotype** |
| --- | --- | --- | --- |
| 1 | M | 13 | Heterozygote |
| 2 | M | 13 | Homozygote Protective |
| 3 | F | 12 | Heterozygote |
| 4 | F | 12 | Homozygote Protective |
| 5 | M | 12 | Heterozygote |
| 6 | M | 12 | Homozygote Protective |
| 7 | M | 14 | Heterozygote |
| 8 | M | 14 | Homozygote Protective |
| 9 | M | 13 | Homozygote Risk |
| 10 | M | 13 | Heterozygote |
| 11 | M | 11 | Homozygote Risk |
| 12 | M | 13 | Homozygote Protective |
| 13 | M | 10 | Homozygote Risk |
| 14 | M | 13 | Homozygote Protective |
| 15 | F | 23 | Homozygote Risk |
| 16 | F | 33 | Homozygote Protective |
| 17 | M | 10 | Homozygote Risk |
| 18 | M | 12 | Homozygote Protective |
| 19 | F | 10 | Homozygote Risk |
| 20 | F | 10 | Heterozygote |
