# Supplementary material for: Predisposition to Childhood Otitis Media and Genetic Polymorphisms within the Toll-Like Receptor 4 (TLR4) Locus
Source: PLoS One. 2015 Jul 15;10(7):e0132551. doi: 10.1371/journal.pone.0132551 (PMC4503307; doi:10.1371/journal.pone.0132551)
Supplement: S4 Table — (DOCX) [file pone.0132551.s007.docx]

**Table S4** Genotyping results for candidate gene study in the Finnish index cohort of 624 affected children and 778 healthy controls. A1 = minor allele, F_A = frequency of minor allele in cases, F_U = frequency of minor allele in controls.

| **SNP** | **Chr** | **Gene** | **A1** | **F_A** | **F_U** | ***P* Value** | **OR** |
| --- | --- | --- | --- | --- | --- | --- | --- |
| rs1801274 | 1 | FCGR2a | A | 0.498 | 0.486 | .533 | 1.05 |
| rs2786098 | 1 | DENND1B, CRB1 | T | 0.186 | 0.188 | .908 | 0.99 |
| rs1554286 | 1 | IL10 | A | 0.176 | 0.193 | .270 | 0.89 |
| rs3021094 | 1 | IL10 | G | 0.083 | 0.088 | .668 | 0.94 |
| rs1800872 | 1 | IL10 | T | 0.240 | 0.243 | .852 | 0.98 |
| rs1800871 | 1 | IL10 | A | 0.235 | 0.242 | .678 | 0.96 |
| rs1800896 | 1 | IL10 | C | 0.448 | 0.462 | .472 | 0.94 |
| rs1800890 | 1 | IL10 | T | 0.348 | 0.371 | .251 | 0.91 |
| rs2020911 | 2 | MSH6 | T | 0.341 | 0.360 | .316 | 0.92 |
| rs330787 | 2 | FBXO11 | A | 0.361 | 0.362 | .968 | 1.00 |
| rs2134056 | 2 | FBXO11 | T | 0.184 | 0.192 | .602 | 0.95 |
| rs3771166 | 2 | IL18R1 | A | 0.386 | 0.405 | .327 | 0.92 |
| rs1800587 | 2 | IL1A | A | 0.322 | 0.342 | .284 | 0.91 |
| rs1588265 | 5 | PDE4D | G | 0.305 | 0.335 | .099 | 0.87 |
| rs2073643 | 5 | SLC22A5 | C | 0.447 | 0.445 | .920 | 1.01 |
| rs2244012 | 5 | RAD50 | G | 0.254 | 0.241 | .420 | 1.08 |
| rs2569190 | 5 | CD14 | A | 0.412 | 0.401 | .591 | 1.04 |
| rs1800750 | 6 | TNFA | A | 0.003 | 0.004 | .689 | 0.75 |
| rs1800629 | 6 | TNFA | A | 0.125 | 0.130 | .688 | 0.95 |
| rs361525 | 6 | TNFA | A | 0.023 | 0.022 | .847 | 1.05 |
| rs3129943 | 6 | C6orf10 | G | 0.278 | 0.279 | .954 | 0.99 |
| rs323917 | 7 | NPSR1 | G | 0.056 | 0.049 | .410 | 1.16 |
| rs323922 | 7 | NPSR1 | C | 0.457 | 0.491 | .088 | 0.87 |
| rs324377 | 7 | NPSR1 | A | 0.468 | 0.504 | .072 | 0.87 |
| rs324396 | 7 | NPSR1 | T | 0.378 | 0.344 | .078 | 1.16 |
| rs3019885 | 8 | SLC30A8 | G | 0.335 | 0.322 | .485 | 1.06 |
| rs2378383 | 9 | TLE4, CHCHD9 | G | 0.178 | 0.201 | .141 | 0.86 |
| rs5030717 | 9 | TLR4 | G | 0.227 | 0.179 | .003 | 1.33 |
| rs10508372 | 10 | LOC338591 | A | 0.068 | 0.072 | .723 | 0.95 |
| rs1800451 | 10 | MBL2 | T | 0.005 | 0.009 | .259 | 0.58 |
| rs7096206 | 10 | MBL2 | G | 0.182 | 0.200 | .254 | 0.89 |
| rs11003125 | 10 | MBL2 | C | 0.419 | 0.444 | .199 | 0.90 |
| rs10762058 | 10 | CTNNA3 | C | 0.240 | 0.236 | .802 | 1.02 |
| rs2735733 | 11 | MUC5B | T | 0.470 | 0.487 | .384 | 0.93 |
| rs7927894 | 11 | C11orf30 | T | 0.297 | 0.290 | .722 | 1.03 |
| rs1701704 | 12 | IKZF4 | G | 0.303 | 0.326 | .214 | 0.90 |
| rs9319321 | 13 | Intergenic | A | 0.369 | 0.386 | .389 | 0.93 |
| rs11071559 | 15 | RORA | T | 0.132 | 0.149 | .213 | 0.87 |
| rs744910 | 15 | SMAD3 | A | 0.486 | 0.484 | .947 | 1.01 |
| rs2305480 | 17 | GSDMB | A | 0.476 | 0.497 | .293 | 0.92 |
| rs11078927 | 17 | GSDMB | T | 0.475 | 0.494 | .350 | 0.93 |
| rs3894194 | 17 | GSDMA | A | 0.417 | 0.402 | .445 | 1.06 |
| rs4815617 | 20 | KIAA1271 | T | 0.054 | 0.056 | .772 | 0.95 |
| rs2284033 | 22 | IL2RB | A | 0.495 | 0.504 | .635 | 0.96 |
