# Supplementary material for: Predisposition to Childhood Otitis Media and Genetic Polymorphisms within the Toll-Like Receptor 4 (TLR4) Locus
Source: PLoS One. 2015 Jul 15;10(7):e0132551. doi: 10.1371/journal.pone.0132551 (PMC4503307; doi:10.1371/journal.pone.0132551)
Supplement: S5 Table — (DOCX) [file pone.0132551.s008.docx]

**Table S5** Follow-up study on *TLR4* in the Finnish index cohort of 624 affected children with RAOM and/or COME and 778 healthy controls. A1 = minor allele, F_A = frequency of minor allele in cases, F_U = frequency of minor allele in controls.

| **SNP** | **Chromosome** | **Position**  **(b37)** | **A1** | **F_A** | **F_U** | **OR** | **95% CI** | ***P Value*** |
| --- | --- | --- | --- | --- | --- | --- | --- | --- |
| rs7875849 | 9 | 120369028 | G | 0.197 | 0.201 | 0.97 | 0.80 – 1.18 | .790 |
| rs1329055 | 9 | 120372882 | T | 0.152 | 0.147 | 1.04 | 0.84 - 1.29 | .716 |
| rs716570 | 9 | 120387228 | A | 0.101 | 0.101 | 0.99 | 0.77 - 1.28 | .943 |
| rs12001662 | 9 | 120406009 | T | 0.490 | 0.487 | 1.01 | 0.87 - 1.18 | .868 |
| rs913615 | 9 | 120418118 | A | 0.456 | 0.472 | 0.94 | 0.81 - 1.09 | .418 |
| rs7039756 | 9 | 120431545 | A | 0.147 | 0.158 | 0.92 | 0.74 - 1.14 | .451 |
| rs10818067 | 9 | 120437061 | T | 0.174 | 0.197 | 0.86 | 0.71 - 1.05 | .134 |
| rs1329060 | 9 | 120438477 | T | 0.281 | 0.228 | 1.33 | 1.11 - 1.58 | .002 |
| rs1329057 | 9 | 120441946 | C | 0.311 | 0.259 | 1.29 | 1.09 - 1.53 | .003 |
| rs11536857 | 9 | 120464136 | T | 0.101 | 0.115 | 0.86 | 0.67 - 1.10 | .239 |
| rs12377632 | 9 | 120472730 | C | 0.305 | 0.332 | 0.88 | 0.75 - 1.04 | .129 |
| rs2770146 | 9 | 120473338 | C | 0.248 | 0.240 | 1.05 | 0.87 - 1.25 | .631 |
| rs4986791 | 9 | 120475602 | T | 0.092 | 0.109 | 0.83 | 0.64 - 1.08 | .160 |
| rs7037117 | 9 | 120483663 | G | 0.278 | 0.285 | 0.96 | 0.81 - 1.14 | .674 |
| rs10759934 | 9 | 120488996 | T | 0.422 | 0.460 | 0.86 | 0.73 - 1.00 | .047 |
| rs2149351 | 9 | 120501644 | T | 0.210 | 0.170 | 1.29 | 1.06 - 1.57 | .010 |
| rs7866830 | 9 | 120502265 | A | 0.174 | 0.192 | 0.89 | 0.73 - 1.08 | .245 |
| rs7870814 | 9 | 120503274 | A | 0.487 | 0.439 | 1.21 | 1.04 - 1.41 | .015 |
| rs10983766 | 9 | 120509848 | G | 0.397 | 0.448 | 0.81 | 0.69 - .95 | .008 |
