# Supplementary material for: Predisposition to Childhood Otitis Media and Genetic Polymorphisms within the Toll-Like Receptor 4 (TLR4) Locus
Source: PLoS One. 2015 Jul 15;10(7):e0132551. doi: 10.1371/journal.pone.0132551 (PMC4503307; doi:10.1371/journal.pone.0132551)
Supplement: S6 Table — (DOCX) [file pone.0132551.s009.docx]

**Table S6** A comparison of data of *TLR4* association to otitis media. A1 = minor allele, fA = frequency of the minor allele, trans/unstrans = transmitted/untransmitted allele in the TDT-test.

| **Haplotype** |  | **FIN all** | |  |  | **FIN index study** | | |  | **FIN replication (205)** | | | | **US, Pittsburgh** | | |  |
| --- | --- | --- | --- | --- | --- | --- | --- | --- | --- | --- | --- | --- | --- | --- | --- | --- | --- |
|  | **A1** | **fA1 case** | **fA1 contr** | **OR** | ***P* Value** | **fA1 case** | **fA1 contr** | **OR** | ***P* Value** | **fA1 case** | **fA1 contr** | **OR** | ***P* Value** | **Trans** | Untrans | OR | P value |
| rs1329060 | T | 0.281 | 0.228 | 1.32 | .002 | 0.281 | 0.228 | 1.33 | .002 | 0.289 |  |  |  | 136 | 18 | 0.79 | .050 |
| rs1329057 | C | 0.311 | 0.259 | 1.30 | .003 | 0.311 | 0.259 | 1.29 | .003 | 0.317 |  |  |  | 168 | 212 | 0.85 | .158 |
| rs5030717 | G | 0.226 | 0.179 | 1.34 | .002 | 0.227 | 0.179 | 1.33 | .003 | 0.223 |  |  |  | 118 | 141 | 0.94 | .654 |
| rs1329060-rs1329057-rs5030717 | T-C-G | 0.227 | 0.180 | 1.34 | .003 | 0.226 | 0.180 | 1.33 | .003 | 0.218 |  |  |  | 77 | 104 | 0.83 | .234 |
|  | T-C-A | 0.055 | 0.049 | 1.16 | .427 | 0.056 | 0.049 | 1.84 | .425 | 0.069 |  |  |  | 17 | 19 | 1.02 | .949 |
|  | C-C-A | 0.029 | 0.031 | 0.95 | .837 | 0.029 | 0.031 | 0.95 | .845 | 0.027 |  |  |  | 37 | 62 | 0.66 | .056 |
|  | C-T-A | 0.689 | 0.740 | 0.77 | .003 | 0.689 | 0.740 | 0.78 | .003 | 0.675 |  |  |  | 591 | 632 | 1.35 | .001 |
| rs1329060-rs1329057 | T-C | 0.282 | 0.229 | 1.32 | .002 | 0.282 | 0.229 | 1.32 | .002 | 0.286 |  |  |  | 97.99 | 129 | 0.84 | .242 |
|  | C-C | 0.029 | 0.030 | 0.96 | .901 | 0.029 | 0.030 | 0.98 | .906 | 0.028 |  |  |  | 43.01 | 68.01 | 0.70 | .080 |
|  | C-T | 0.689 | 0.741 | 0.77 | .003 | 0.689 | 0.741 | 0.78 | .003 | 0.681 |  |  |  | 608 | 645 | 1.38 | .008 |
| rs1329057-rs5030717 | C-G | 0.227 | 0.181 | 1.32 | .004 | 0.201 | 0.181 | 1.32 | .004 | 0.217 |  |  |  | 93 | 115 | 0.91 | .530 |
|  | C-A | 0.085 | 0.078 | 1.10 | .493 | 0.085 | 0.078 | 1.11 | .483 | 0.097 |  |  |  | 70 | 88.02 | 0.89 | .518 |
|  | T-A | 0.689 | 0.741 | 0.77 | .003 | 0.689 | 0.741 | 0.77 | .003 | 0.680 |  |  |  | 635 | 706 | 1.12 | .034 |
|  |  | **UK Trios (all together)** | | |  | **Unvaccinated UK Trios (216 informative trios)** | | | | **Vaccinated UK Trios (216 informative trios)** | | | | **US, Portland** | | | |
|  | **A1** | **Trans** | **Untrans** | **OR** | ***P* Value** | **Trans** | **Untrans** | **OR** | ***P* Value** | **Trans** | **Untrans** | **OR** | ***P* Value** | **fA1 case** | **fA1 contr** | **OR** | ***P* Value** |
| rs1329060 | T | 273 | 274 | 1.00 | .966 | 71 | 77 | 0.92 | .622 | 50 | 43 | 1.16 | .468 | 0.085 | 0.148 | 0.53 | .059 |
| rs1329057 | C | 347 | 349 | 0.99 | .940 | 98 | 104 | 0.94 | .673 | 54 | 54 | 1.00 | 1.000 | 0.125 | 0.185 | 0.63 | .113 |
| rs5030717 | G | 222 | 214 | 1.04 | .702 | 59 | 61 | 0.97 | .855 | 44 | 32 | 1.38 | .169 | 0.061 | 0.099 | 0.59 | .179 |
| rs1329060-rs1329057-rs5030717 | T-C-G | 225 | 214.5 | 1.05 | .616 | 61 | 65.26 | 0.94 | .705 | 44 | 31 | 1.42 | .133 | 0.055 | 0.099 | 0.53 | .116 |
|  | T-C-A | 68.15 | 76.59 | 0.89 | .483 | 19.78 | 19.73 | 1.00 | .993 | 8 | 14 | 0.57 | .201 | 0.030 | 0.050 | 0.60 | .334 |
|  | C-C-A | 113.1 | 128.3 | 0.88 | .327 | 34 | 42.17 | 0.81 | .350 | 13 | 21.27 | 0.61 | .158 | 0.040 | 0.037 | 1.08 | .884 |
|  | C-T-A | 373.7 | 357.4 | 1.05 | .549 | 111.9 | 10.4 | 1.12 | .432 | 58.77 | 56.5 | 1.04 | .832 | 0.870 | 0.815 | 1.52 | .148 |
| rs1329060-rs1329057 | T-C | 265 | 252 | 1.05 | .568 | 68 | 68 | 1 | 1 | 50 | 39 | 1.28 | .244 | 0.125 | 0.185 | 0.53 | .113 |
|  | C-C | 104 | 119 | 0.87 | .315 | 32 | 37 | 0.87 | .547 | 11 | 21 | 0.52 | .077 | 0.915 | 0.852 | 1.08 | .059 |
|  | C-T | 336 | 331 | 1.02 | .847 | 96 | 90 | 1.07 | .660 | 54 | 53 | 1.02 | .923 | 0.960 | 0.963 | 1.59 | .884 |
| rs1329057-rs5030717 | C-G | 215.5 | 197.5 | 1.09 | .376 | 58 | 58 | 1 | 1 | 42 | 28 | 1.50 | .094 | 0.055 | 0.098 | 0.53 | .116 |
|  | C-A | 166.5 | 185.5 | 0.90 | .311 | 52 | 57 | 0.91 | .632 | 18 | 34 | 0.53 | .027 | 0.070 | 0.087 | 0.80 | .556 |
|  | T-A | 34.5 | 339.5 | 1.00 | .969 | 99 | 96 | 1.03 | .830 | 54 | 52 | 1.04 | .846 | 0.870 | 0.814 | 1.52 | .148 |
